# A patient who recovered from post-COVID myalgic encephalomyelitis/chronic fatigue syndrome: a case report

**DOI:** 10.1186/s13030-022-00260-3

**Published:** 2023-02-28

**Authors:** Takakazu Oka

**Affiliations:** Department of Psychosomatic Medicine, International University of Health and Welfare Narita Hospital, 852 Hatakeda, Narita, Chiba 286-8520 Japan

**Keywords:** Post-COVID ME/CFS, COVID-19, Myalgic encephalomyelitis, Chronic fatigue syndrome, Long COVID, SARS-CoV-2, Post-viral fatigue syndrome

## Abstract

**Background:**

Some patients infected with the severe acute respiratory syndrome coronavirus 2 (SARS-CoV-2) complain of persistent fatigue, dyspnea, pain, and cognitive dysfunction. These symptoms are often described as “long COVID”. Whether a patient with long COVID might develop myalgic encephalomyelitis/chronic fatigue syndrome (ME/CFS) is of interest, as is the treatment and management of ME/CFS in a post-COVID patient. Here I report a patient, who, after an infection with SARS-CoV-2, developed ME/CFS and recovered after treatment.

**Case presentation:**

The patient was a previously healthy 55-year-old woman who worked as a nurse and became ill with COVID-19 pneumonia. She then presented with severe fatigue, post-exertional malaise, dyspnea, pain, cognitive dysfunction, tachycardia, and exacerbation of fatigue on physical exertion, which persisted for more than 6 months after her recovery from COVID-19 pneumonia. She was bedridden for more than half of each day. The patient was treated from multiple perspectives, which included (1) instructions on eating habits and supplements; (2) cognitive and behavioral modifications for coping with physical, emotional, and cognitive fatigue; (3) instructions on conditioning exercises to improve deconditioning due to fatigue and dyspnea; and (4) pharmacotherapy with amitriptyline and *hochuekkito*, a Japanese herbal (*Kampo*) medicine. The patient made a complete recovery after completing the prescribed regimen and was able to return to work as a nurse.

**Conclusions:**

To the best of my knowledge, this is the first detailed report on a patient infected with SARS-CoV-2 followed by long COVID with the signs/symptoms of ME/CFS who recovered after treatment. I hope this case report will be helpful to health care practitioners by its presentation of some of the therapeutic options for alleviating disabling signs/symptoms in patients with post-COVID ME/CFS.

## Background

Some patients infected with the severe acute respiratory syndrome coronavirus 2 (SARS-CoV-2) complain of persistent fatigue, dyspnea, pain, or cognitive dysfunction [1, 2]. Signs/symptoms that persist for longer than several months beyond the acute phase of infection are often termed “long COVID”. No consensus treatment for long COVID has as yet been established; however, the importance of holistic assessment and multidisciplinary care and management has been emphasized [3, 4].

Of note, similar signs/symptoms can be observed in patients with myalgic encephalomyelitis/chronic fatigue syndrome (ME/CFS). ME/CFS is a debilitating disease that is characterized by a constellation of symptoms such as severe fatigue, post-exertional malaise (PEM), cognitive dysfunction, and pain. If these symptoms persist for more than 6 months, they are diagnosed as ME/CFS [5, 6]. The pathophysiological mechanisms of ME/CFS are not fully understood. However, it is well known that viral infections, including SARS-CoV-1 [7, 8], can trigger the development of ME/CFS. Therefore, it has become of great interest if a patient who was infected with SARS-CoV-2 and developed long COVID could eventually develop ME/CFS. It is also important to investigate if long COVID and post-infectious ME/CFS share the same pathophysiological mechanisms, and, even more important, to establish treatments for both conditions [9]. Here, I report on a woman with long COVID who was infected with SARS-CoV-2 and developed severe fatigue and other debilitating problems that persisted for more than 6 months, thus satisfying the diagnostic criteria for ME/CFS. She recovered after treatment for her ME/CFS that was based on multiple perspectives.

## Case presentation

A 55-year-old woman was referred to our department for treatment of severe fatigue that developed after SARS-CoV-2 infection. She had worked as a nurse for more than 20 years. In April 2020, a cluster of infections with SARS-CoV-2 occurred in her hospital. On April 19, she developed a fever of 38.3℃. A polymerase chain reaction (PCR) test of a nasopharyngeal swab was positive for SARS-CoV-2. She took a leave of absence and was almost completely bedridden at home. On May 9, she was hospitalized in the Department of Respiratory Medicine because of exacerbation of her dyspnea, which was diagnosed as COVID-19 pneumonia. She received ciclesonide (200 μg, twice a day) for 14 days. After confirmation of a negative PCR test for COVID-19, she was discharged on May 28. However, she continued to have severe fatigue and dyspnea at rest despite a percutaneous arterial oxygen saturation of 98%. Thereafter, her dyspnea at rest gradually improved. However, she fatigued easily and complained of palpitations. Her heart rate increased to 120 beats per minute (bpm) after a short walk.

Because she had continued weakness and lack of stamina, she decided to retire. On a follow-up visit to the Department of Respiratory Medicine in June, her signs and symptoms were diagnosed as the sequelae of a coronavirus infection, i.e. long COVID. In August, she was examined in the Department of Neurology, which did not find any abnormalities that would account for her signs and symptoms. A neurologist suspected ME/CFS and referred her to our department for treatment on December 2.

On her first visit to our department, the patient complained of severe fatigue and PEM after physical and cognitive exertion and spent about 60% of her day in bed. She also complained of weakness, dyspnea, palpitations, pharyngeal pain, arthralgia, and exacerbation of a sensation of heat around her neck and head on physical exertion. When her malaise increased, her sore throat worsened. She initially complained of insomnia and nonrestorative sleep; however, these symptoms gradually resolved. Her complaints satisfied the 1994 Fukuda case definition of CFS [5]. Before her visit to our department, her complaints to other physicians also satisfied the 2003 Canadian clinical case definition of myalgic encephalomyelitis (ME)/CFS [10]. However, because her sleeping problems gradually resolved, she did not quite satisfy the Canadian clinical case definition at the time of her visit to our department.

The patient had a history of allergic rhinitis. Her past and family histories were negative for psychiatric disorders. She had a negative history for alcohol consumption and smoking. Her physical examination was unremarkable except for an axillary temperature of 37.2° C. Results of laboratory testing of a peripheral blood sample were unremarkable. She was not anemic and had normal thyroid function. Her serum cortisol level was 5.34 μg/dL at 16:00 h. Her electrocardiogram was normal, and cardiac echography showed preserved left ventricular systolic function. During an active standing test, she complained of palpitations and worsening fatigue. She showed orthostatic hypertension, was negative for orthostatic hypotension, and has slight orthostatic tachycardia (Table 1).

**Table 1:** Clinical test values before and after undergoing the treatment protocol

	1st visit	Last visit
Axillary temperature (℃)	37.2	36.6
Fatigue level (NRS)	5	0
BFI score	7.0	0.5
TMT-A (sec)	45	35
Active standing test SBP/DBP (HR)		
Recumbent (baseline) After standing	130/74 (84)	124/79 (90)
2 min	140/89 (98)	143/88 (96)
3 min	142/90 (100)	137/90 (94)
4 min	142/86 (98)	141/84 (92)
5 min	131/88 (100)	136/85 (95)
Average after standing	138.8/88.3 (99.0)	139.3/86.8 (94.3)
Change by standing	8.8/14.3 (15.0)	15.3/7.8 (4.3)

Clinical parameters were compared on the first and last visits to our department

Fatigue level was measured by 2 methods: the NRS, with 10 being the most severe fatigue that she could imagine and 0 being no fatigue; and the Japanese version of the BFI score. During the active standing test, the SBP (mm Hg), DBP (mm Hg), and HR (beats per minute) were recorded every min for 5 min. (Values were not obtained for the first min after standing at the first visit; therefore, the table only shows the values at 2, 3, 4, and 5 min after standing.) Average after standing: average values from 2 to 4 min. Changes by standing: average values after standing – baseline values

*BFI* Brief fatigue inventory, *DBP* Diastolic blood pressure, *HR* Heart rate, *NRS* Numerical rating scale, *SBP* Systolic blood pressure, *TMT-A* Japanese version of the trail-making test A

Her fatigue score on the numerical rating scale (NRS) was 5: the maximum fatigue score is10 and an absence of fatigue is scored 0. On the brief fatigue inventory (BFI), which was developed to assess the degree of fatigue of cancer patients [11], her score was 7.0, which corresponds to severe fatigue. The Japanese version of the trail making test-A (TMT-A) was used to evaluate her cognitive function [12, 13]. Her completion time was 45 s (mean time for healthy subjects her age is 33.7 ± 6.2 s), indicating impaired cognitive function compared to healthy participants of her age [13]. Her score on the Center for Epidemiologic Studies Depression Scale, a self-rating questionnaire for evaluating depression, was 9, indicating that she was not depressed.

### Treatment

The patient’s disabling symptoms included the following. (1) Severe fatigue at rest and PEM, even after minimal physical or mental exertion; she spent more than 50% of the day in bed. After physical exertion such as going to the supermarket, she became bedridden for several hours to days. After mental exertion such as paperwork for retirement, she had difficulty concentrating and understanding and had to read the same sentence repeatedly. (2) Weakness, dyspnea, tachycardia, and exacerbation of a sensation of heat around her neck and head on physical exertion. Even after her resting dyspnea improved, she experienced these symptoms during and after physical exertion such as when walking or standing upright. When she felt hot, her axillary temperature increased up to 37.5℃. (3) Dull pain. She first complained of hip pain because of osteoarthritis of the left hip joint, which was initially relieved by nonsteroidal anti-inflammatory drugs (NSAIDs) or resting. After the infection, she developed generalized dull pain that was not alleviated by NSAIDs or rest. These symptoms led to her retirement.

**Fig. 1:**
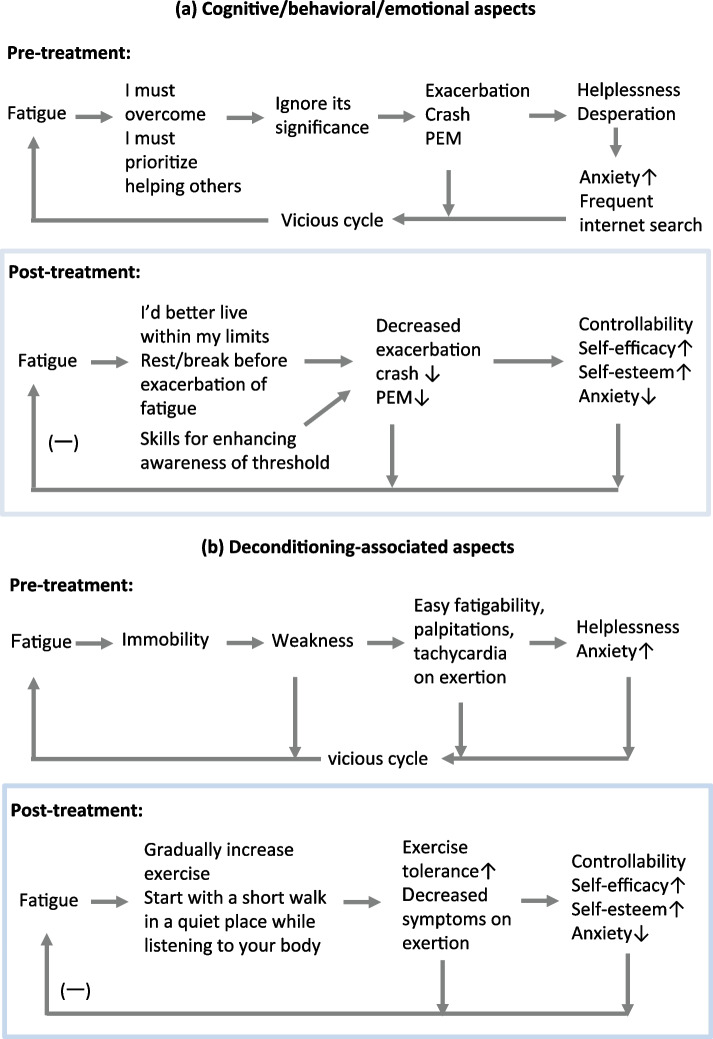
Therapeutic strategies for changing the cognitive, behavioral, emotional, and deconditioning-associated factors that might have impeded the recovery of this patient from post-COVID ME/CFS. The fatigue associated with post-COVID ME/CFS has been thought to be derived from the biological mechanisms associated with this condition. However, as the duration of the post-infection period lengthens, fatigue itself or the natural recovery from this condition might be modulated by a variety of factors. In our patient, two factors were thought to be associated with her ME/CFS. The schematic diagrams, although speculative, illustrate how these pre-treatment factors might have led to a vicious cycle and impeded her recovery and how the interventions facilitated her post-treatment recovery. **a** Cognitive/behavioral/emotional aspects: Before treatment, the patient’s coping strategies were based on maladaptive beliefs and her limited knowledge of the characteristics of the fatigue associated with this disease, i.e., physical, emotional, or cognitive exertion easily induced crashing and PEM. After treatment, her adaptive coping efforts were based on her acceptance of the condition and her increased knowledge of how to manage the fatigue associated with this condition. **b** Deconditioning-associated aspects. (-) indicates amelioration after treatment. ME/CFS: myalgic encephalomyelitis/chronic fatigue syndrome; PEM: post-exertional malaise

She was treated from multiple perspectives according to our usual treatment regimen for patients with ME/CFS [14–16] (Table 2). (1) Instruction on eating habits and supplements. After her infection with SARS-CoV-2, the patient changed to a vegetable-based diet and began daily ingestion of ascorbic acid (1000 mg/day). She was advised to maintain these habits to minimize the potentially harmful effects of oxidative stress. (2) Cognitive and behavioral modifications of daily life for adaptive self-management, with the following interventions (evaluation of her current coping strategy against fatigue; understanding and acknowledgement of her beliefs and feelings behind current strategies; encouragement for acquisition of new coping strategies for management of symptoms, including “self-pacing”; enhancement of awareness of thresholds resulting in exacerbation of fatigue, exhaustion, and PEM; initiation of relaxing and meditative-relaxing movements; minimization of cognitive fatigue caused by habitual rumination of negative thoughts; improvement in self-esteem and self-efficacy; development of a habit of adaptive coping and appreciation of its value) (Fig. 1a).

**Table 2:** Summary of the therapeutic strategies that were used for this patient

**(1) To minimize the potentially harmful effects of oxidative stress**
She was advised to eat a vegetable-based diet and to take ascorbic acid daily
**(2) To maximize adaptive coping and self-management skills**
The patient’s initial coping strategies against fatigue were evaluated. We endeavored to understand and acknowledge her beliefs and feelings that led to the initial strategies; encouraged the acquisition of new coping strategies, e.g., “self-pacing”; encouraged awareness of thresholds resulting in “crashing” and PEM; taught meditative-relaxing movements; taught minimization of cognitive fatigue due to habitual rumination of negative thoughts; and encouraged improvement in self-esteem and self-efficacy (see Fig. 1a for details)
**(3) To reverse deconditioning and to improve deconditioning-associated symptoms**
She was advised to start exercising gradually, taking precautions (see Fig. 1b for details)
**(4) Pharmacotherapy**
Amitriptyline (10 mg) and *hochuekkito* (7.5 g) were prescribed

Because of the patient’s limited energy and memory, written explanations for how to achieve her cognitive and behavioral modifications were provided. The explanations included outlining the self-help strategies and precautions in daily life for ME/CFS patients. She was asked to follow the advice as much as possible. She was encouraged to consult me with any questions and confusion about the prescribed regimen. These interventions were not conducted by introducing her to cognitive behavioral therapists, but were done during medical treatment for her cognitive and physical limitations on the day of her visit. (3) After her dyspnea and fatigue at rest were improved, exercise was gradually added to her regimen to help her recover from physical deconditioning, while at the same time avoiding exhaustion and PEM (Fig. 1b). (4) Oral amitriptyline (10 mg before sleep) and *hochuekkito*, a Japanese herbal (*Kampo*) medicine (7.5 g before every meal) were prescribed.

### Clinical course

After her first visit to our department, the patient began to take her prescribed medication and to perform the activities of daily life while being aware of the precautions she needed to take. At her second visit 2 weeks later, she reported that her fatigue, pain, and low-grade fever had started to decrease when she was at rest. She also said that she learned the idea of “idling” (i.e., ruminating on negative thoughts at rest) and found it very helpful because she noticed that reduced idling actually improved her cognitive fatigue. At the third visit (6 weeks later), she reported that her low-grade fever and sensation of heat had disappeared. At the fourth visit (11 weeks later), she said she was in good shape and her fatigue was almost zero when she was at rest; however, she did not have enough endurance for shopping. Therefore, she was instructed to gradually increase her exercise to improve deconditioning. At the sixth visit (21 weeks later), she said she had become able to practice the activities of daily living without medication and that her physical and mental condition had recovered to almost the same level as that before she became ill. She reported that she was planning to return to work, and her treatment was terminated. Table 1 shows her clinical test values on her first and last visits. In addition to improvement of her axillary temperature, subjective level of fatigue, BFI score, and the TMT-A result, the average heart rate (HR) from 2 to 5 min after standing (mean ± standard deviation, from 99.0 ± 1.2 bpm to 94.3 ± 1.7 bpm, *P* = 0.015, paired t-test) and the change of HR by standing (from 15.0 ± 1.2 bpm to 4.3 ± 1.7 bpm, *P* = 0.002, paired t-test) were also significantly reduced.

## Discussion and conclusions

To the best of my knowledge, this is the first report of a patient who developed ME/CFS after SARS-CoV-2 infection and recovered after treatment for her ME/CFS. The therapeutic strategy employed was based on my clinical experience in treating patients with ME/CFS [14–16]. To date, there is no single treatment that can cure every patient with ME/CFS, which might be accounted for by the fact that many abnormalities are thought to be involved in this illness. Therefore, my patients are usually treated from multiple perspectives to resolve as many abnormalities as possible with the aim of providing treatment that exerts a synergistic effect. For this case, I focused on four aspects (Table 2).

The first was to minimize the effects of oxidative stress. Oxidative stress has been suggested to be involved in both long COVID [17] and ME/CFS [18]. Therefore, it is reasonable to hypothesize that antioxidant therapy would be beneficial. For example, intravenous administration of high-dose ascorbic acid (> 7.5 g, in most cases) has been reported to alleviate fatigue, inability to concentrate, and the pain of patients with long COVID [17]. Although the dose of ascorbic acid the patient ingested was 1.0 g/day, her vegetable-based diet and regular supplementation of ascorbic acid would be helpful for avoiding the potentially harmful effects of oxidative stress and aid in alleviating her symptoms. Recently, the gut microbiota is reported to be associated with the ME/CFS pathophysiology, including neuroinflammation and ME/CFS symptoms [19]. It is also possibile that changes in dietary habits that affected the gut microbiota was responsible for improved symptoms.

The second aspect was to maximize adaptive coping and self-management skills (Fig. 1a). First, her coping strategies against fatigue and her beliefs and feelings behind them were evaluated. She believed that she should not give in to fatigue but had to endure and overcome it. This idea seemed to derive, at least partly, from the fact that when she was young she was athletic and trained herself to overcome fatigue without complaining in order to become stronger. She used this strategy not only when she felt fatigued but also when she had pain and cognitive problems. It was obvious that these coping strategies resulted in exhaustion and PEM and exacerbated her symptoms. Furthermore, because of her concerns for other people she prioritized helping others instead of resting, even when she felt fatigue. Her beliefs and feelings might have made it difficult for her to accept adaptive coping strategies. Eventually, the vicious cycle of repeatedly working to exhaustion led to feelings of helplessness, worthlessness, and desperation, as well as exacerbation of her illness. Therefore, while acknowledging her beliefs and previous coping strategies, she was first provided written instructions that included precautions to follow and information on how to develop adaptive coping strategies. It was emphasized that fatigue is not something to overcome but rather to be managed by resting “not when” she became fatigued but “before” she started feeling fatigue (or worsening of fatigue), in order to live with a limited amount of energy. Second, the importance of reducing the repetition of negative thoughts, worry, and self-blame, i.e., “idling”, in order to avoid cognitive fatigue was emphasized. Third, she was encouraged to increase her ability to be aware of her threshold for exhaustion or PEM and to attain the habit of “listening to her body”, e.g., by recording in an "energy-saving passbook" and writing in a "fever-fatigue diary" [16]. The passbook and diary were suggested with the aim of shifting her focus of attention from “symptoms that varied every day” to “inner bodily wisdom that helped her to recover” [20] and to change her mindset/attitudes from “worry and helplessness” to “that is something I can handle”, both of which may lead to improvement in her self-esteem and self-efficacy.

The third aspect was reverse deconditioning (Fig. 1b). After the patient’s fatigue and dyspnea at rest improved, she was encouraged to gradually start exercising, such as taking short, slow walks to improve the vicious cycle of fatigue-deconditioning-fatigue. When she started to exercise, she was given some precautions to keep in mind. One was to start walking in a quiet place and avoid noisy and crowded places. Another was to be mindful of her thresholds and to be conscious of and to make wise choices based on the following advice. “Do such exercises (types and duration) that result in your feeling pleasantly fatigued during and after them, just as you felt when you were healthy. However, avoid such exercises (types and duration) that result in your feeling unwell during and after them.”

The role of physical exercise in the treatment of ME/CFS remains debatable, whereas the importance of multidisciplinary rehabilitation is stressed in treating long COVID [3, 4]. As was suggested for the treatment of long COVID, it was thought that treating the deconditioning of this patient was important because she had been completely bedridden for longer than a week because of pneumonia. Her signs/symptoms such as dyspnea, weakness, and tachycardia on exertion might have been derived, at least partly, from deconditioning. Previous studies have demonstrated that deconditioning occurs even after several days of bed rest, and atrophy of diaphragmatic muscle fibers can be observed within several days of diaphragmatic inactivity due to mechanical ventilation [21]. Her weakness, dyspnea, and tachycardia on exertion might not have been caused by deconditioning alone. Orthostatic intolerance and dysautonomia, especially postural orthostatic tachycardia, are common in patients with long COVID and ME/CFS [22, 23]. Cumulative evidence suggests that such autonomic dysfunction is associated with autoantibodies to β adrenergic and muscarinic cholinergic receptors [22, 23]. To improve such dysautonomia, pharmacotherapy such as α-1 receptor agonists or β-blockers was also to be considered as well as instructions on exercise.

The fourth aspect was pharmacotherapy. Amitriptyline and *hochuekkito* were prescribed for this patient. Amitriptyline has anxiolytic and sleep-inducing properties and activates the inhibitory nociceptive system as well as acting as an anti-depressant. It also has an antihistaminergic effect. *Hochuekkito* is a Japanese herbal medicine for improving “*ki-kyo*”, i.e., a low-energy state, according to traditional Japanese (*Kampo*) medicine [24, 25]. *Hochuekkito* has been found to ameliorate fatigue in patients with such chronic diseases as cancer, chronic obstructive pulmonary disease, and ME/CFS [26, 27]. It has also been found to improve hypocortisolemia and decreased heart rate variability [28] and, furthermore, to inhibit neuroinflammation [29]. Together, amitriptyline and *hochuekkito* may have acted synergistically to ameliorate the patient’s ME/CFS.

After the treatment protocol was completed, the patient’s axillary temperature returned to the normal range and the sensation of heat disappeared. The NRS of the patient’s fatigue decreased from 5 to 0, and the BFI score decreased from 7.0 to 0.5. Her cognitive function as assessed by the TMT-A also improved. The tachycardia that occurred during standing also improved.

There are several limitations to this case report. First, it is not possible to identify which elements of the prescribed regimen were the most effective for her recovery. Although the treatments seem to have worked synergistically, further studies are necessary. Second, it might be that the apparent beneficial effects of the treatment protocol were actually a reflection of the natural process of recovery. However, the patient had been almost bed-ridden for longer than 6 months and started to feel better as early as 2 weeks after the start of treatment. Therefore, even if she was in the process of natural recovery, the interventions may have facilitated her recovery. Third, I could not fully assess the patient’s change in cognitive function. The TMT-A is a test to assess focused attention. Because patients with ME/CFS also complain of memory impairment, additional comprehensive assessments of cognitive function should be included in a future study. Despite the limitations of this report, I believe that this is the first case report that has described in detail the time course of a patient who developed long COVID and ME/CFS after being infected by SARS-CoV-2 and who recovered because of treatment based on multiple perspectives.

## Data Availability

Data sharing is not applicable.

## Abbreviations

BFI: Brief fatigue inventory
bpm: Beats per minute
CD: Chronic disease
CFS: Chronic fatigue syndrome
COVID: Coronavirus disease
ME/CFS: Myalgic encephalomyelitis/chronic fatigue syndrome
NRS: Numerical rating scale
NSAIDs: Nonsteroidal anti-inflammatory drugs
SARS-CoV-2: Severe acute respiratory syndrome coronavirus 2
TMT-A: Trail making test-A
